# Naval Appointments

**Published:** 1858-09

**Authors:** 


NAVAL APPOINTMENTS.—The Board of Naval Surgeons recently convened in this city, consisted of Surgeons Greene, President; Ruschenberger and Foltz, members; and Passed Assistant Surgeon Howell, Recorder. Twenty-seven candidates presented themselves for examination, of whom the following gentlemen were selected as qualified for the post of Assistant Surgeons in the United States Navy: Drs. Bertolette, of Pa.; Leach, of N. H.; Christian, of Va.; Megee, of Pa.; Gibbs, of N. J.; Burnett, of Pa.; and King, of Pa.

